# Differential dynamic microscopy of bidisperse colloidal suspensions

**DOI:** 10.1038/s41526-017-0027-7

**Published:** 2017-08-30

**Authors:** Mohammad S. Safari, Ryan Poling-Skutvik, Peter G. Vekilov, Jacinta C. Conrad

**Affiliations:** Department of Chemical and Biomolecular Engineering, University of Houston, Houston, TX 77204-4004 USA

## Abstract

Research tasks in microgravity include monitoring the dynamics of constituents of varying size and mobility in processes such as aggregation, phase separation, or self-assembly. We use differential dynamic microscopy, a method readily implemented with equipment available on the International Space Station, to simultaneously resolve the dynamics of particles of radius 50 nm and 1 μm in bidisperse aqueous suspensions. Whereas traditional dynamic light scattering fails to detect a signal from the larger particles at low concentrations, differential dynamic microscopy exhibits enhanced sensitivity in these conditions by accessing smaller wavevectors where scattering from the large particles is stronger. Interference patterns due to scattering from the large particles induce non-monotonic decay of the amplitude of the dynamic correlation function with the wavevector. We show that the position of the resulting minimum contains information on the vertical position of the particles. Together with the simple instrumental requirements, the enhanced sensitivity of differential dynamic microscopy makes it an appealing alternative to dynamic light scattering to characterize samples with complex dynamics.

## Microscopy: All shapes and sizes

A way to measure the motion of tiny particles of different sizes on the International Space Station is developed by researchers in the USA. Jacinta Conrad, Peter Vekilov, and their colleagues from the University of Houston demonstrate that this can be achieved using a technique called differential dynamic microscopy. Colloidal systems can contain particles of a wide variety of sizes, all moving at different rates. Conrad and the team demonstrate that differential dynamic microscopy has the ability to resolve the motion of polystyrene particles of two different sizes suspended together in water. The equipment required for their experiment is readily available on the International Space Station, and so the approach could help to better understand how potentially a microgravity environment affects complex dynamics in a variety of systems.

## Introduction

Microgravity provides a unique environment in which to investigate the physics of transport processes such as diffusion, convection, and conduction. These processes affect structure in systems featuring sub-microscale constituents, including bacterial biofilms,^1, 2^ protein crystals,^3, 4^ and complex fluids.^5^ Monitoring dynamics on these length scales in microgravity is expected to generate fundamental insight into the physics controlling structural evolution. One traditional method for characterizing dynamics at the microscale, dynamic light scattering (DLS),^6^ is already available on the International Space Station (ISS)^7^ but is restricted by the detector frame rate to characterize slow motions. An intriguing alternative is provided by recent enhancements to the Light Microscopy Module (LMM) on the ISS, which increased the time resolution of image acquisition and imparted confocal imaging capabilities. These advances make it possible to access faster dynamics across a broad range of samples but require methods to obtain dynamics from microscopy time series images.

For microscale structures that can be directly visualized on an optical microscope, dynamics can be extracted from a time-series of microscopy images via image-processing algorithms. The numerical aperture on a standard light microscope, however, limits the size of resolvable structures to typically greater than 150 nm using the smallest feasible wavelength of light. Although advanced super-resolution microscopy methods can in principle lower this size limit significantly,^8^ low time resolution and stringent instrumentation requirements limit their immediate adoption on the ISS. Hence there remains a need for methods that can be readily implemented on a standard microscope with relatively simple equipment, compatible with the strict demands of the space environment.

Differential dynamic microscopy (DDM),^9^ an extension of heterodyne near-field scattering^10^ and one of an emerging family of digital Fourier techniques,^11^ is a flexible, powerful, and readily-implemented method to probe microscale dynamics. In DDM, the microscale dynamics of a sample are extracted from the decorrelation of intensity fluctuations evaluated from a time series of difference images.^12^ This method has two key advantages: first, it has minimal instrumentation requirements, and, second, it can access smaller wavevectors and hence larger length scales than conventional DLS setups. Thus, DDM has been used to characterize the dynamics of dispersed nanoparticles^13–15^ and bacteria,^16, 17^ as well as colloidal^18^ and protein^19^ condensates. Further, DDM has been extended to imaging modes beyond brightfield, including fluorescence,^12^ confocal,^20^ and darkfield^21^ microscopy. Hence this method offers new flexibility and capability to investigate complex dynamic phenomena using microscopy.

The simple instrumental requirements of DDM allow it to be implemented on the ISS to enable novel probes of dynamics in microgravity. As one example, gravity significantly alters dynamic processes controlled by a single mobile species, including colloidal aggregation and phase separation^22^ or multiscale self-assembly.^23^ In suspensions containing mobile constituents of varying size and mobility, gravity may play an even more significant role. Indeed, many physical processes are driven by differences in the dynamics of distinct constituents, such as suspension phase behavior,^24–28^ flow-induced margination,^29, 30^ or the self-organization of active matter.^31–34^ Studies of these processes in microgravity are expected to elucidate their complex physics; the varied nature of these systems requires a powerful, flexible, and easily-implementable method, such as DDM. Although DDM has been extensively applied to systems featuring relatively simple dynamics described by a single characteristic relaxation time and to mixtures with Gaussian distribution of relaxation times such as protein aggregates, its application to systems featuring nonuniform complex dynamics has been limited to samples with multi-step relaxations.^35, 36^

Here, we demonstrate a new application of DDM: the ability to resolve dynamics in a complex mixture containing two sizes of particles, using equipment comparable to that in the LMM on the ISS. We formulate dilute mixtures of polystyrene particles of radius 50 nm and 1 micron at different ratios of the large-to-small fraction at modest total volume fractions of *ϕ* ~ 10^−3^, at which both species freely diffuse. The 50 nm particles are too small to be resolved using standard optical methods. Using DLS and DDM, we measure the particle diffusivities in the mixtures. Whereas DLS is not sufficiently sensitive to resolve the dynamics of both species at these concentrations, DDM successfully measures the diffusivities of both large and small particles. The enhanced sensitivity of DDM derives from the preferential forward scattering of large objects. The scattered light from the large particles generates interference patterns that affect the amplitude of the dynamic correlation function. We show that this amplitude is non-monotonic and corresponds to the interference pattern, and thus may be used to characterize their average axial position. We anticipate that this approach can be applied to time series of images acquired on the LMM and in other space experiments—enhancing the time resolution and providing new insights into microscale and nanoscale dynamics in microgravity.

## Results and discussion

### Dynamic light scattering

As a control experiment, we measured the diffusivities of particles of radius 50 nm and 1 µm, respectively, using DLS. In suspensions containing particles of uniform size, the intermediate scattering function *f*(*q*,*t*) could be fitted to a single exponential,

1 $$f(q,t) = \exp \left( { - \frac{t}{{{\tau _S}}}} \right)$$

where the time scale *τ*_*S*_ was related to the particle diffusivity via *D*_*S*_ = 1/*q* ^2^ *τ*_*S*_. The measured diffusivities of the small (4.3 ± 0.1 μm^2^ s^−1^) and large (0.20 ± 0.02 μm^2^ s^−1^) particles were in good agreement with the diffusivities predicted from the Stokes–Einstein equation using the nominal radii (4.3 μm^2^ s^−1^ and 0.21 μm^2^ s^−1^).

**Fig. 1:**
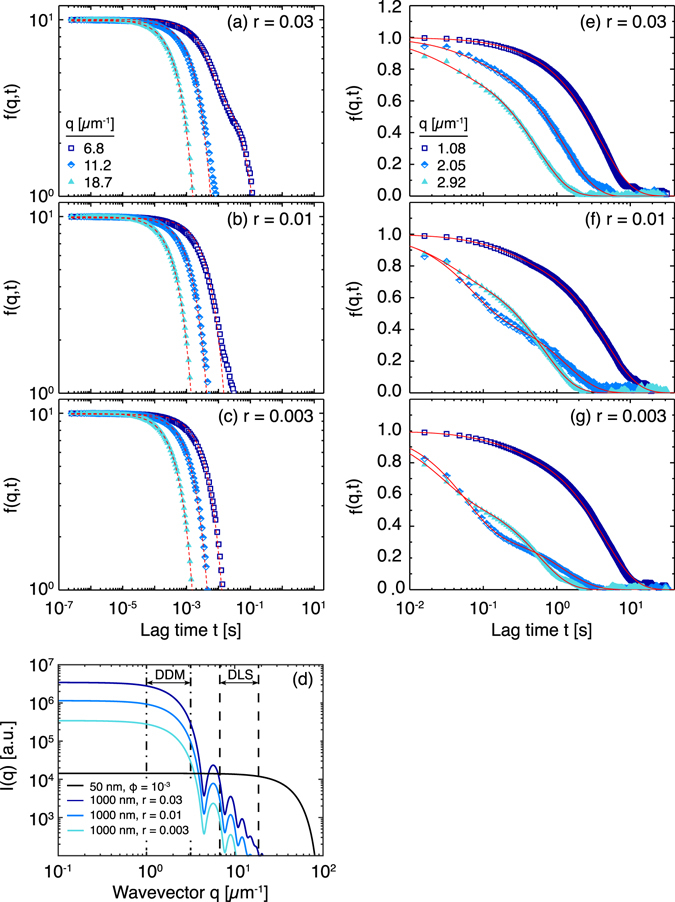
**a**–**c** Intermediate scattering function *f*(*q*,*t*) as a function of lag time *t* measured for bidisperse mixtures of particles of radius 50 nm and 1 μm formulated at a large-to-small volume fraction ratio *r* of **a** 0.03, **b** 0.01, and **c** 0.003 at wavevectors of *q* = 6.8 μm^−1^ (30°, *squares*), 11.2 μm^−1^ (50°, *diamonds*), and 18.7 μm^−1^ (90°, *triangles*). *Red lines* indicate fitting functions: Eq. 2 for *r* = 0.03 and *q* = 6.8 μm^−1^ and Eq. 1 otherwise. **d** Predicted scattering intensity *I*(*q*) for small particles at *ϕ* = 10^−3^ and large particles at volume fraction ratios of *r* = 0.03, 0.01, and 0.003 as a function of wavevector *q* using standard equations for hard spheres.^50^ The range of wavevectors probed by DLS and DDM are indicated by *dashed* and *dash-dotted lines*, respectively. **e**–**g** Intermediate scattering function *f*(*q*,*t*), extracted from DDM measurements, as a function of lag time *t* measured for bidisperse mixtures of particles of radius 50 nm and 1 μm formulated at large-to-small volume fraction ratios *r* of **a** 0.03, **b** 0.01, and **c** 0.003. For each ratio, data were analyzed over the wavevector range 0.98 μm^−1^ < *q* < 3.01 μm^−1^; the figure shows representative correlation functions obtained for wavevectors *q* = 1.08 μm^−1^ (*squares*), 2.05 μm^−1^ (*diamonds*), or 2.92 μm^−1^ (*triangles*). *Red lines* indicate fits to Eq. 4

To test the ability of DLS to measure dynamics of both species in a bidisperse mixture, we formulated samples containing a constant volume fraction of small particles, *ϕ*_*S*_ = 10^−3^, and added large particles at various concentrations to obtain volume fraction ratios of *r* = *ϕ*_*L*_/*ϕ*_*S*_ = 0.03, 0.01, and 0.003. The intermediate scattering functions *f*(*q*,*t*), measured at three scattering angles, exhibited distinct shapes depending on the concentration of large particles. At the highest concentration of large particles (*r* = 0.03) and the lowest scattering wavevector (*q* = 6.8 µm^−1^), *f*(*q*,*t*) exhibited a second shoulder at long lag times (Fig. 1a); by contrast, no second shoulder was apparent at higher angles (e.g., for *q* = 18.7 µm^−1^ in Fig. 1a) or at lower concentrations of large particles (e.g., at *q* = 6.8 µm^−1^ and *r* = 0.003 in Fig. 1c).

For bidisperse suspensions, the choice of an appropriate fitting model was determined by the scattering properties of the particles. The large particles used in these experiments were Mie scatterers^37^: the Mie parameter *x* for a particle of radius *a*_*L*_ = 1 µm interacting with light of wavelength *λ* = 632.8 nm in water (refractive index *n* = 1.33) was *x* = 2*πa*_*L*_ *n*/*λ* = 13.2, much larger than the Rayleigh threshold^38^ *x* = 1. The Mie parameter for the small particles was *x* = 0.66, slightly below this threshold. In the Mie regime, the scattering intensity is anisotropic with preferential forward scattering at low angles. Therefore, the contribution of both particles to the intermediate scattering function was angle-dependent and concentration-dependent. To capture these physics, we used two fitting forms: a single exponential decay when the scattering from small particles dominated and a double exponential decay when scattering from both populations was significant. For scattering experiments at *r* = 0.003 and 0.01, the correlation functions at all three scattering angles were fitted with a single exponential function (Eq. 1). The diffusivities calculated from the fitted time scale, *D*_*S*_ = 1/*q* ^2^ *τ*_*S*_, reflected the rate of diffusion of small particles over the length scale 2π/*q* and were in good agreement with that from the unary control experiment (Table 1). At a higher large-particle ratio *r* = 0.03, the intermediate correlation function exhibited a second shoulder at the lowest wavevector (*q* = 6.8 μm^−1^) indicative of two populations of diffusing particles. For *r* = 0.03, we fitted the intermediate correlation functions at *q* = 6.8 μm^−1^ to the sum of two exponential functions

2 $$f(q,t) = {f_S}\exp \left( { - \frac{t}{{{\tau _S}}}} \right) + {f_L}\exp \left( { - \frac{t}{{{\tau _L}}}} \right)$$

where *τ*_*S*_ and *τ*_*L*_ are the characteristic diffusion times of 50 nm and 1 µm particles, respectively, and *f*_*S*_ and *f*_*L*_ = 1−*f*_*S*_ are proportional to the amplitude of the scattered signal produced by each particle population. Again, the diffusion coefficient for each particle species was extracted from its characteristic diffusion time via *D*_*S*,*L*_ = 1/*q* ^2^ *τ*_*S*,*L*_. The calculated diffusivities were larger than but comparable to those from the unary control measurements (Table 1); *D*_*L*_, in particular, was significantly larger. The inability to accurately detect the large particles across the accessible range of wavevectors prohibited the use of DLS to characterize minority-large bidisperse suspensions at low volume fractions. Hence, we explored alternate methods for characterizing dynamics in these samples.

**Table 1:** Diffusivities obtained from dynamic light scattering measurements for unary (top two rows) and bidisperse (labeled with volume fraction ratio *r*) samples

				Diffusion coefficient [μm^2^/s]	
	Radius [nm]	*ϕ*	*q* [μm^−1^]	*D*_S_	*D*_L_
	50	10^−3^		4.3 ± 0.1	^a^
***r*** ***=*** *ϕ*_**L**_ **/** *ϕ*_**S**_	1000	10^−5^		^a^	0.20 ± 0.02
0.003	50	10^−3^	6.8	3.8 ± 0.2	^b^
	1000	3 × 10^−6^	11.2	4.1 ± 0.1	^b^
			18.7	4.3 ± 0.1	^b^
0.01	50	10^−3^	6.8	4.5 ± 0.4	^b^
	1000	1 × 10^−5^	11.2	4.3 ± 0.1	^b^
			18.7	4.5 ± 0.1	^b^
0.03	50	10^−3^	6.8	4.9 ± 0.3	0.30 ± 0.05
	1000	3 × 10^−5^	11.2	4.1 ± 0.3	^b^
			18.7	4.1 ± 0.1	^b^

Error bars are the standard deviation from 10 independent runs. The Stokes-Einstein diffusivities are 4.3 and 0.21 μm^2^/s for small and large particles, respectively

^a^ measurements made on unary samples lacking this particle population

^b^ unable to resolve second particle population

### Differential dynamic microscopy

To evaluate the sensitivity of DDM to distinguish particles of two different sizes, we performed DDM measurements on the same series of samples. In the DDM theory, the structure function Δ(*q*;*t*) is related to the intermediate scattering function *f*(*q*,*t*) via

3 $$\Delta \left( {q;t} \right) = A\left( q \right)\left( {1 - f(q,t)} \right) + B\left( q \right)$$

where *A*(*q*) depends on the optical transfer function of the imaging system and on the scattering properties of objects, and *B*(*q*) captures any noise introduced into the system.^9, 12^ For a population of monodisperse scatterers at low concentration, *f*(*q*,*t*) is commonly fit to a single exponential decay (Eq. 1). In samples with more complex dynamics, such as those featuring multiple relaxation timescales,^35, 36^ a single exponential decay cannot be applied.

Here, our goal was to determine the extent to which the sizes of particles in a bidisperse mixture could be resolved. Because DDM accesses a lower range of wavevectors than our DLS setup, the scattering intensity from the large particles is more pronounced than in DLS (Fig. 1d). Thus, we expected to observe two decays in the DDM signal for bidisperse mixtures, corresponding to the rate of diffusion for each particle size. At the highest concentration of large particles and at the lowest wavevectors, the signal from the large particles dominates; the small particles still contribute to the intensity at lower volume fraction ratios and higher wavevectors. To capture the contributions from both particles, we globally fit all relaxations across the wavevector range to the sum of two single-exponential functions with a weighting function *f*_*S*_(*q*) to describe the relative contribution from each particle population. This fitting form has five fitting parameters at each wavevector: *τ*_*S*_(*q*), *τ*_*L*_(*q*), *A*(*q*), *B*(*q*), and *f*_*S*_(*q*). To reduce the number of independent fitting parameters, we noted that the ratio of the decay rates of the large and small particles should be constant across the range of wavevectors, even as the relative scattering contribution from each was modulated by the anisotropic scattering of the particles. For bidisperse mixtures, we therefore implemented a global fitting process and fit to the structure function

4 $$\\ 	\Delta \left( {q;t} \right) = \\ 	 A\left( q \right)\left[ {1 - \left( {{f_S}\left( q \right)\exp \left( { - \frac{t}{{{\tau _L}(q)/{f_r}}}} \right) + {f_L}\left( q \right)\exp \left( { - \frac{t}{{{\tau _L}\left( q \right)}}} \right)} \right)} \right] + B(q)$$

where *τ*_*L*_(*q*) is the relaxation time of 1 µm particles at the wavevector *q*; the weighting functions *f*_*S*_(*q*) and *f*_*L*_(*q*) = 1−*f*_*S*_(*q*) describe the contribution of small (50 nm) and large (1 μm) particles, respectively, to the scattering intensity at *q*; and *f*_*r*_ is the ratio of relaxation times of large and small particles, which is independent of *q* and hence was globally fit. Although the ratio *f*_*r*_ is known for these particles from the control experiments, *a priori* knowledge of the particle sizes is not required to use this functional form. This functional form exploits the full dynamic range of the DDM technique to generate a more robust non-linear fitting methodology and thereby accurately measure the diffusivities of both particles in a bidisperse mixture.

We obtained *f*(*q*,*t*) for each wavevector from series of difference images.^19^ In contrast to the intermediate scattering functions measured at higher angles using DLS, the DDM *f*(*q*,*t*) clearly show non-exponential decays over 0.98 μm^−1^ < *q* < 3.01 μm^−1^ for all values of *r* examined here (Fig. 1e–g). This *q*-range is narrower than that accessed by us in earlier measurements using similar equipment^13–15^ and is limited by the dynamics of the particles relative to the rate of image acquisition (Fig. S1 in Supplementary Information). For *q* < 0.98 μm^−1^, the upper plateau was not reached by the maximum lag time at which we obtained enough independent measurements for statistics, 35 s (2200 frames), which was set by the frame rate and camera buffer. For *q* > 3.01 μm^−1^, the frame rate (63 fps) was insufficient to resolve the diffusive relaxation time scale of the smaller particles. Nonetheless, the data in Fig. 1e–g indicate that DDM can resolve particle dynamics in a bidisperse mixture.

The intermediate plateaus observed in the DDM intermediate scattering functions (Fig. 1e–g) resulted from the large particle size ratio $\left(\frac{{{a_L}}}{{{a_S}}} \approx 20 \right)$. When $\frac{{{a_L}}}{{{a_S}}} < 10$, the two relaxations will not be well separated. Instead, *f*(*q*,*t*) will resemble a stretched exponential decay, characteristic of a dynamic process with a distribution of relaxation rates.^13, 35^ Careful analysis of the residuals of a stretched exponential and a double exponential fit can distinguish between these decays.^39^ For mixtures of particles of comparable size, the distribution of particle sizes will manifest as a polydispersity term, which can be captured by fitting a cumulant form to the intermediate scattering function.^19^

**Fig. 2:**
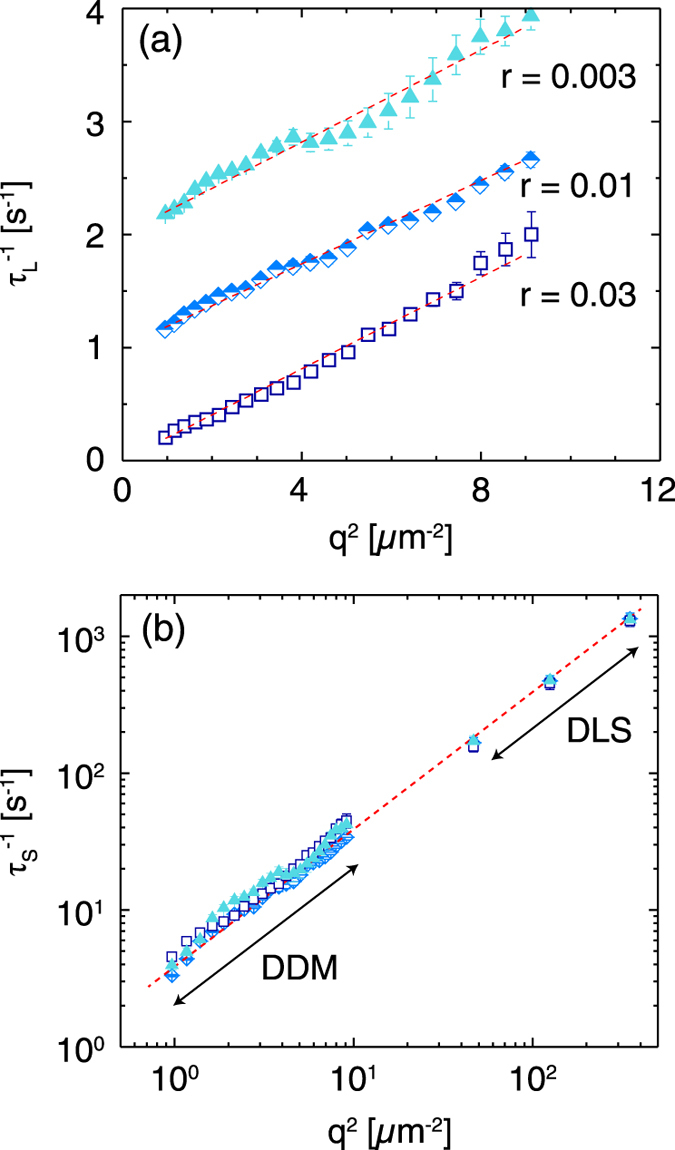
**a** Inverse of the large-particle time scale $\tau _L^{ - 1}$ as a function of the square of the wavevector *q* ^2^ for bidisperse mixtures of particles of radius 50 nm and 1 μm formulated at large-to-small volume fraction ratios *r* = 0.03 (*squares*), 0.01 (*diamonds*), and 0.003 (*triangles*). Data at *r* = 0.01 and *r* = 0.003 are offset by one and two unit increments on the *y* axis, respectively, for clarity. **b** Comparison of DLS and DDM inverse time scales for the small particles as a function of *q* ^2^. Data at low wavevectors are acquired in a bidisperse mixture using DDM; data at higher wavevectors are acquired in unary solutions using DLS. *Dashed red lines* in **a** and **b** indicate linear fits

To confirm that DDM yields quantitative information on the dynamics of bidisperse mixtures, we examined the *q*-dependence of *τ*_*L*_, which was not resolvable with DLS. The inverse of this time scale, $\tau _L^{ - 1}$, scaled linearly with *q* ^2^ over the given range of wavevectors, indicating that the particle motion was diffusive (Fig. 2). Furthermore, the diffusion coefficient of the particles, extracted from the slope of a linear fit of $\tau _L^{ - 1}$ as a function of *q* ^2^, was in agreement with that obtained on a unary sample of large particles with DLS and with DDM. Finally, we compared relaxation times of the small particles *τ*_*S*_ = *τ*_*L*_/*f*_*R*_ extracted from the DDM fits on bidisperse suspensions to those obtained on unary suspensions in DLS. Within fitting errors, we obtained good agreement between the diffusion coefficients obtained in unary (control) and in bidisperse suspensions (Table 2). Thus, DDM accurately measures the dynamics of both particles in a bidisperse suspension, beyond the capabilities of DLS. This enhanced sensitivity of DDM arises because we study a small population of large scatterers in a suspension of smaller scatterers (a relevant limit for early-stage aggregation, as one example). In the opposite limit, where the small particles are the minority species in a bidispersed mixture, DLS would be a more sensitive technique because the scattering from the large population of large scatterers would overwhelm that from the small particles in DDM.

**Table 2:** Diffusivities obtained from differential dynamic microscopy experiments for unary (top rows) and bidisperse (labeled with volume fraction ratio *r*) samples

			Diffusion coefficient [μm^2^/s]	
	Radius [nm]	*ϕ*	*D*_S_	*D*_L_
	50	10^−3^	^a^	0.22 ± 0.01
*r* *=* *ϕ*_L_/*ϕ*_s_	1000	10^−5^	4.1 ± 0.1	^a^
0.003	50	10^−3^	4.3 ± 0.3^b^	0.20 ± 0.01
	1000	3 × 10^−6^		
0.01	50	10^−3^	4.0 ± 0.2^b^	0.19 ± 0.01
	1000	1 × 10^−5^		
0.03	50	10^−3^	4.4 ± 0.4^b^	0.20 ± 0.01
	1000	3 × 10^−5^		

Error bars are numerical uncertainty from fitting functions. Stokes-Einstein diffusivities are 4.3 and 0.21 μm^2^/s for small and large particles, respectively

^a^ measurements made on unary samples lacking this particle population

^b^ calculated from the experimentally-measured large-particle diffusivity using the globally-fit size ratio *f*_*r*_

### DDM signal generation in bidisperse suspensions

DDM is a heterodyne scattering method, in which the scattered light interferes with the transmitted light. The structural information extracted from heterodyne near field scattering depends on the sample-to-detector distance.^10^ DDM, by contrast, accurately captures the dynamics of a sample regardless of the sample-to-detector distance^12^ because the dynamic information is encoded in the time dependence of the intermediate scattering function rather than in the signal amplitude.

**Fig. 3:**
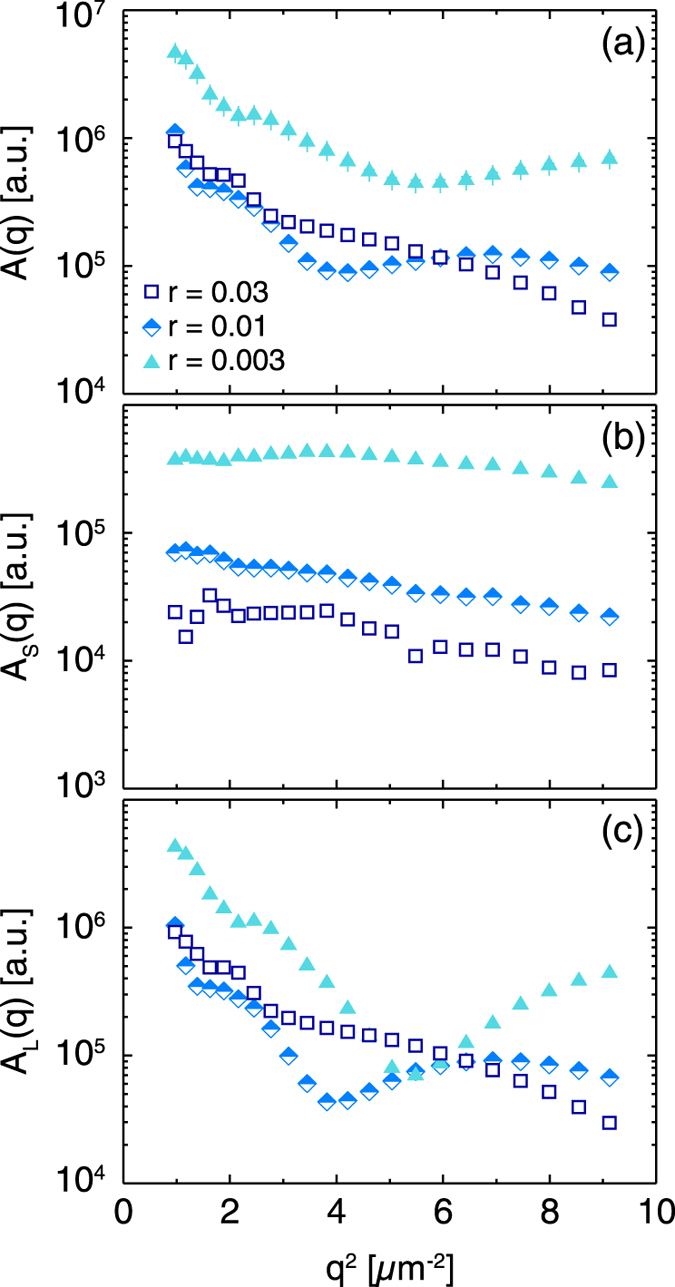
**a** DDM signal amplitude *A*(*q*) as a function of wavevector squared *q* ^2^ for bidisperse mixtures of particles of radius 50 nm and 1 μm at varying volume fraction ratios *r*. **b** and **c** describe the contributions to signal intensity from small and large particles, *A*_*S*_(*q*) and *A*_*L*_(*q*), respectively

In DDM, the signal amplitude *A*(*q*) is the product of the optical transfer function, which depends on the imaging set up, and the scattering pattern of the particles.^12^ For the bidisperse samples at *r* = 0.01 and 0.003, *A*(*q*) was non-monotonic (Fig. 3a). To investigate the origins of this non-monotonicity, we extracted the contributions to the DDM signal from the small and large particles (Fig. 3b, c) by multiplying *A*(*q*) by the relative contributions *f*_*S*_(*q*) and *f*_*L*_(*q*), respectively. Whereas the intensity of the small particles decayed monotonically with increasing wavevector at all *r*, the intensity of the large particles exhibited non-monotonic oscillations at *r* = 0.01 and 0.003. Similar oscillations are seen in *I*(*q*) for the large particles (Fig. 1d), but at higher wavevectors than those probed with DDM; over the *q*-range probed by DDM, *I*(*q*) of the large particles is predicted to decrease monotonically. Thus, the oscillations in the DDM amplitude were not caused by the scattering intensity, but by the optical transfer function acting on the large particles.

**Fig. 4:**
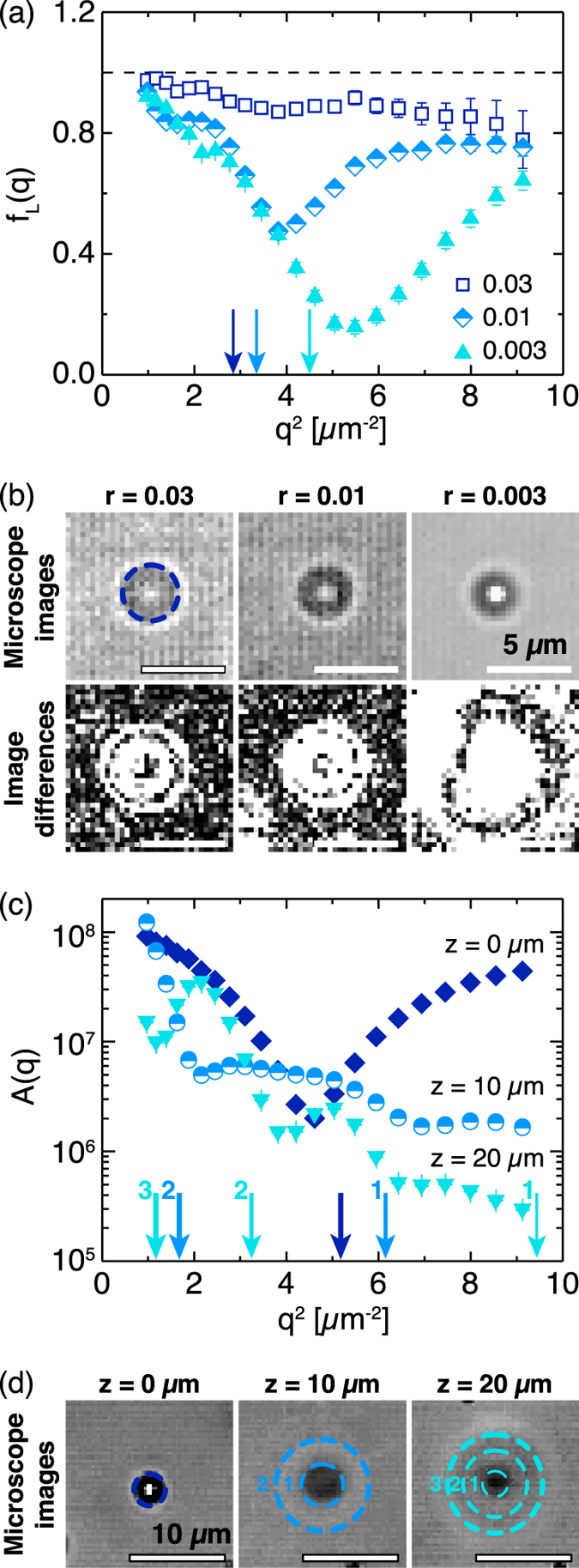
**a** Relative contribution to the DDM signal from the large particles *f*_*L*_(*q*) as a function of the square of the wavevector *q* ^2^. *Arrows* indicate predicted minima from the diameter of the interference rings. The incident light intensity and sample thickness were kept constant between all samples but the condenser numerical aperture was set to ~0.1 for *r* = 0.03 and 0.01 and to ~0.15 for *r* = 0.003 to improve the signal-to-noise ratio. **b** Examples of interference rings seen in microscope images (*top*) and image differences (*bottom*) at a lag time *τ* = 1.6 s for each sample. *Dashed circle* indicates the diameter of the interference ring. **c** DDM signal amplitude *A*(*q*) for large particles segregated via sedimentation to the bottom of a glass sample chamber and imaged at various heights *z* above the plane of the segregated particles. *Arrows* indicate the predicted minima from the diameter of interference rings. **d** Examples of interference rings in microscope images for segregated particles imaged at the same heights. *Dashed circles* indicate the diameters of the interference rings corresponding to *arrows* in **c**. Microscope images and image differences were modified to increase brightness and contrast for clarity

For monodisperse samples of small particles with *r*/*λ* < 1, the optical transfer function for the DDM signal decays exponentially with *q* with a rate set by a roll-off wavevector *q*_ro_.^12^ By contrast, large objects with *r*/*λ* ≫ 1 act as phase objects, introducing oscillations to the optical transfer function and consequently to *A*(*q*).^12, 37^ To identify the positions of the non-monotonic oscillations in the large-particle signal, we examined the relative scattering intensity of the large particles *f*_*L*_(*q*) (Fig. 4). This measure isolates the oscillations from the exponential decay of the optical transfer function to more accurately identify the local minima. Although the incident light intensity and sample thickness were held constant for all *r*, we varied the condenser aperture from ~0.1 for *r* = 0.03 and 0.01 and to ~0.15 for *r* = 0.003 to improve the signal-to-noise ratio. Because of this change in experimental conditions, we avoid direct comparisons between *f*_*L*_(*q*) for the three samples. Nevertheless, all three samples exhibited pronounced minima in the relative intensities. For *r* = 0.03 and 0.01, the primary minima occurred at *q* ^2^ ≈ 4 μm^−2^ and for *r* = 0.003, at *q* ^2^ ≈ 5.5 μm^−2^. We attribute these minima to the interference patterns from the large particles present in the microscope images and in the series of difference images (Fig. 4b).

In a heterodyne geometry, interference patterns are complex due to the presence of both scattered and transmitted fields. The best examples of interference patterns are holograms,^40–43^ in which the scattered field interferes with the transmitted light, and diffraction patterns,^44, 45^ in which the scattered field interferes with itself. These patterns typically depend on the particle radius and the distance *z* between a particle and the image plane, which ideally can be resolved to 50 nm.^41, 44^ The DDM algorithm, however, averages the scattering signal from multiple particles at varying axial positions. Additionally, the particles diffuse vertically and this motion changes the position of the rings over time. Thus, the minima present in *f*_*L*_(*q*) are smeared without the ideal axial resolution of diffraction-based or hologram-based particle tracking methods. Nevertheless, we compared the position of the minima *f*_*L*_(*q*_min_) to the diameter *d* = 2*π*/*q*_min_ of the interference rings in the captured original images, calculated as the boundary between the dark ring and the outer light ring (*dashed circle* in Fig. 4b). While we do not attempt to predict changes in the position of *f*_*L*_(*q*_min_) between samples, the close agreement between the diameters of the rings and *f*_*L*_(*q*_min_) (Table 3) suggests that it is the interference patterns that lead to the non-monotonic changes in the DDM signal intensity. This argument implies that *f*_*L*_(*q*_min_) depends on particle size, incident wavelength, and distance of particles from focal plane but is in principle independent of particle concentration.

**Table 3:** Measured interference pattern diameter and predicted *q*_min_ from microscope images

*r* *=* *ϕ*_L_/*ϕ*_S_	Ring diameter *d* [μm]	Predicted *q*_min_ = 2*π*/*d* [μm^−1^]	Actual *q*_min_ [μm^−1^]
0.003	3.0 ± 0.7	2.1 ± 0.5	2.3 ± 0.1
0.01	3.5 ± 0.7	1.8 ± 0.4	2.0 ± 0.1
0.03	3.7 ± 0.7	1.7 ± 0.3	2.0 ± 0.1

Error in ring diameter equivalent to ±2 pixels. Error in *q*_min_ is equal to the *q*-resolution of DDM

To test this hypothesis, we performed a proof-of-concept experiment on unary suspensions of large particles that were segregated via sedimentation to the bottom of the glass sample chamber. The particles remained diffusive on the glass surface, but hydrodynamic interactions with the surface reduced their diffusivity compared to that in the bulk (Figs. S2 and S3, Supplementary Information). Focusing the lens at different distances *z* above the plane of the segregated particles, we controlled the distance between the particles and the image plane. The amplitude of the DDM signal *A*(*q*) depended on the vertical position (Fig. 4c) and the locations of the minima corresponded nearly quantitatively with observed changes in the interference patterns (Fig. 4d). Thus, the amplitude *A*(*q*) of the dynamic structure factor Δ(*q*;*t*) can, in principle, be used to extract information about the axial position of large particles, similar to diffraction-based^44^ or holographic^40, 43^ particle tracking methods in three dimensions. Although difficult to predict the interference patterns *a priori* due to the distribution of wavelengths and the spherical wavefront of the incident light in a DDM experiment, similar experiments could be performed at smaller height steps to calibrate the observed interference patterns. This calibration would allow determination of the height by matching the measured *A*(*q*) to the calibrated *A*(*q*).

Future experiments could exploit a designed separation of time scales (by tuning particle size, solution viscosity, and/or density difference) to characterize multiple dynamic processes simultaneously. For example, in microgravity environments where sedimentation is minimal, experiments could separate aggregation^18^ and conjugation^46^ from the motion of individual particles. In terrestrial experiments, in-plane diffusion could be characterized with DDM, and sedimentation velocities could be calculated tracking the changes in *A*(*q*) over a series of movies acquired over extended times.

### DDM in microgravity

The simplicity of this method well suits it for experimental tests of microgravity effects on the dynamics of colloid suspensions and biological solutions on the ISS. The LMM facility on the ISS is equipped with 63 × oil immersion lenses (Leica, 11506350 and 11506062) and a 100 × oil immersion lens (Leica, 11506372) with numerical aperture of 1.4, similar to that used in our experiments. The LMM is equipped with an Imperx Bobcat B2020 camera with frame rates of 51 and 100 fps and a field of view of 125 × 177 μm^2^ for the 63 × lens or 77 × 105 μm^2^ for the 100 × lens. Wide-field epi-illumination is provided by a metal halide arc lamp-coupled to a solid core light pipe, whose intensity can be tuned to improve image quality by an epi aperture block. LMM was recently updated with a GIU confocal system and fluorescent filter cubes including 436 nm (blue) Filter P/N D436/10X (Chroma) and 546 nm (green) Filter P/N 11504010 (Leica), which additionally permit confocal and fluorescent DDM in addition to brightfield DDM.

## Conclusions

We show that DDM can be used to obtain information about the dynamics of multiple constituents even at very dilute concentrations not detectable with DLS. In addition, DDM on bidisperse mixtures offers a new capability: inferring the axial position of the larger scatterers through physics similar to that underlying diffraction-based and hologram-based particle tracking methods. We believe the ability to characterize both axial position and in-plane dynamics can be exploited to measure dynamics on different time scales, such as sedimentation velocity and diffusion. The simplicity of DDM, and the availability of compatible facilities on the ISS, indicates that this method can be applied to obtain dynamical information across a broad range of systems studied in microgravity. For example, ground-based studies use DDM to measure concentration fluctuations in binary^47^ and ternary^48^ fluid mixtures; hence this method can be readily applied to the wealth of image time-series data available from extant microgravity studies on similar systems.^49^

## Methods

### Materials

Fluoro-Max Dyed Red Fluorescent polystyrene particles with radii *a*_*L*_ = 1 µm and *a*_*S*_ = 50 nm (dispersity <5%, as reported by the manufacturer, consistent with measures in Fig. S4) were purchased from Thermo Fisher Scientific. The nanoparticles were packaged as aqueous suspensions at a concentration of 1% solids by weight, which contained a trace amount of surfactant to inhibit particle aggregation. The refractive index of the nanoparticles was 1.59 and their density was 1.06 g cm^−3^.

Samples for DLS and DDM experiments were prepared by diluting dispersions of nanoparticles from the as-received concentration (1 wt%) with deionized water that was filtered with 0.2 μm polyethersulfone syringe filters (Sterlitech). To minimize aggregation and ensure uniform dispersion, all samples were bath sonicated for 10 s prior to sample preparation. In these experiments, we fixed the volume fraction of the small particles at *ϕ*_*S*_ = 10^−3^ and prepared solutions with volume fraction ratio of large to small particles of *r* = *ϕ*_*L*_/*ϕ*_*S*_ = 0.003, 0.01, and 0.03 (i.e., *ϕ*_*L*_ = 3 × 10^−5^ for r = 0.03, *ϕ*_*L*_ = 10^−5^ for r = 0.01, and *ϕ*_*L*_ = 3 × 10^−6^ for *r* = 0.003).

### Dynamic light scattering

DLS data were collected with an ALV goniometer equipped with a He–Ne laser (wavelength *λ* = 632.8 nm) and an ALV-5000/EPP Multiple tau Digital Correlator (ALV-GmbH, Langen, Germany). For DLS experiments, samples were loaded into cylindrical cuvettes of diameter 10 mm. To minimize contamination, all cuvettes were washed with DI water prior to loading into the light scattering instrument. We collected the light scattered at a fixed angle *θ* and a temperature of 20 °C for 110 s and repeated this measurement ten times at each of three scattering angles θ = 30°, 50°, and 90°, corresponding to wavevectors $q = \left( {\frac{{4\pi n}}{\lambda }} \right)\sin \left( {\theta /2} \right)$ of 6.84, 11.18, and 18.7 µm^−1^, where *n* = 1.331 is the refractive index of water. From the scattered intensity as a function of time, the normalized intensity-intensity correlation function *g*_2_(*q*,*t*) = 〈*I*(*t*_0_ + *t*)*I*(*t*_0_)〉/〈*I*(*t*_0_)〉^2^ was calculated for each wavevector *q* at lag times *t* ranging from 0.1 μs to 10 s.

### Differential dynamic microscopy

Samples for DDM were sealed in glass chambers constructed from cover glasses. Two (22 × 22)-mm^2^ cover glasses (thickness 0.19–0.23 mm, Fisherbrand), separated laterally by ∼10 mm, were attached to a rectangular cover glass of dimensions 48 × 65 mm^2^ (thickness 0.13–0.17 mm, Gold Seal) using an epoxy-based adhesive (Devcon). A (22 × 22)-mm^2^ cover glass was then centered on top of the two cover glasses to create an open chamber. One side of the chamber was sealed with epoxy. Particle suspensions were introduced into the chamber through the open side, which was then sealed with epoxy.^14^ We assumed that the thickness of this chamber was ~160 μm. For DDM data collection, particle suspensions were imaged on a Leica inverted microscope attached to a 63 × oil immersion objective lens (NA = 1.4) using an 8-bit camera (AOS Technologies AG) at room temperature (≈20 °C). The numerical aperture of the microscope condenser was 0.4 (NA = *D*/*f*, where *f* = 100 mm is the focal length of the tube lens and *D* = 40 mm is the diameter of exit light), which introduces too much incoherency to accurately implement DDM.^12^ Hence to optimize the imaging conditions for DDM experiments, we manually reduced the condenser aperture to ~0.1 for *r* = 0.03 and 0.01 and to ~0.15 for *r* = 0.003, estimated by measuring the ratio of the average intensity of the images to the intensity of images acquired with the condenser fully open. For each sample, we recorded two series of 4200 images of size 480 × 640 pixels^2^ (pixel size: 0.305 μm, leading to an image size of 146.4 × 195.0 μm^2^) at a frame rate of 63 frames s^−1^.

To extract the dynamics of each diffusive population from micrographs, a DDM algorithm was implemented as described in ref 12. Briefly, images separated by a fixed lag time *t* were subtracted to obtain the intensity difference, Δ(*x*,*y*;*t*) = *I*(*x*,*y*,*t*_0_ + *t*)−*I*(*x*,*y*;*t*_0_), where *I*(*x*,*y*;*t*_0_) was the intensity at position (*x*,*y*) measured at time *t*_0_; in these experiments, lag time *t* ranged from 0.0158 to 34.9 s. The image subtraction produced a speckle pattern, which could be analyzed to extract information about fluctuations in concentration on different length scales. We applied a two-dimensional fast Fourier transform to each image in the image difference series, generated its 2D power spectrum Δ(*u*_*x*_, *u*_*y*_; *t*), and averaged over all starting times *t*_0_ at a constant lag time *t*. Because the particles were spherical and could freely diffuse in all directions, the 2D power spectrum was isotropic. Hence, we azimuthally averaged Δ(*u*_*x*_, *u*_*y*_; *t*) to generate the DDM structure function Δ(*q*;*t*), where $q \equiv 2\pi \sqrt {u_x^2 + u_y^2}$.

In theory, the range of wavevectors accessible in DDM was determined by the experimental geometry. The minimum accessible wavevector was estimated to be *q* = 2*π*/*l* ≈ 23 μm^−1^, where *l* = 195 μm was the largest dimension of the images. Similarly, the maximum accessible wavevector was estimated to be $q = 2\pi \sin \left( {{\theta _{{\rm{max}}}}} \right)/\lambda \approx 10.6$ μm^−1^, where *θ*_max_ ≈ 68° was the maximum accessible angle of the lens and *λ* ≈ 550 nm was the wavelength of the incident light.^12^ In practice, neither geometric limit was accessible for our bidisperse mixtures, due to the time scales of the particle dynamics and practical limitations set by the number of frames (set by the acquisition camera). At low *q*, the dynamics typically did not fully decorrelate and the upper plateau of the structure function was not reached, resulting in poor fitting; at high *q*, the frame rate was insufficiently fast to capture the diffusive relaxation time of the 50 nm particles (Supplementary Information Fig. S1). Hence, we restricted the *q*-range to 0.98 μm^−1^ < *q* < 3.01 μm^−1^ in these experiments.

### Data availability

The datasets generated and analyzed during the current study are available in the Open Science Framework repository, https://osf.io/93cb5/ (doi: 10.17605/OSF.IO/93CB5). Additional movie files are also available from the corresponding author on reasonable request.

## Electronic supplementary material

Supplementary Information

Supplementary Movie M1 100 frames of a brightfield microscopy movie acquired for a sample with small particle volume fraction of *ϕ*_S_ = 10^−3^ and a large-to-small ratio of *r* = 0.03. Movie acquired at 63 fps; playback is at 25 fps. Scale bars are 50 μm.

Supplementary Movie M2 100 frames of a brightfield microscopy movie acquired for a sample with small particle volume fraction of *ϕ*_S_ = 10^−3^ and a large-to-small ratio of *r* = 0.01. Movie acquired at 63 fps; playback is at 25 fps. Scale bars are 50 μm.

Supplementary Movie M3 100 frames of a brightfield microscopy movie acquired for a sample with small particle volume fraction of *ϕ*_S_ = 10^−3^ and a large-to-small ratio of *r* = 0.003. Movie acquired at 63 fps; playback is at 25 fps. Scale bars are 50 μm.
