# Supplementary material for: Differential dynamic microscopy of bidisperse colloidal suspensions
Source: NPJ Microgravity. 2017 Aug 30;3:21. doi: 10.1038/s41526-017-0027-7 (PMC5577322; doi:10.1038/s41526-017-0027-7)
Supplement: Supplementary file 1 — Supplementary Information [file 41526_2017_27_MOESM1_ESM.pdf]

Differential Dynamic Microscopy of Bidisperse Colloidal Suspensions

Mohammad S. Safari, Ryan Poling-Skutvik, Peter G. Vekilov,* and Jacinta C. Conrad*
Department of Chemical and Biomolecular Engineering, University of Houston, Houston, TX 77204-4004
*Corresponding authors: jcconrad@uh.edu, vekilov@uh.edu

Supplemental Movie M1_100fr_r_0.03.avi. 100 frames of a brightfield microscopy movie acquired for a sample with small particle volume fraction of $\phi_s = 10^{-3}$ and a large-to-small ratio of $r = 0.03$. Movie acquired at 63 fps; playback is at 25 fps. Scale bars are 50 µm.

Supplemental Movie M2_100fr_r_0.01.avi. 100 frames of a brightfield microscopy movie acquired for a sample with small particle volume fraction of $\phi_s = 10^{-3}$ and a large-to-small ratio of $r = 0.01$. Movie acquired at 63 fps; playback is at 25 fps. Scale bars are 50 µm.

Supplemental Movie M3_100fr_r_0.003.avi. 100 frames of a brightfield microscopy movie acquired for a sample with small particle volume fraction of $\phi_s = 10^{-3}$ and a large-to-small ratio of $r = 0.003$. Movie acquired at 63 fps; playback is at 25 fps. Scale bars are 50 µm.

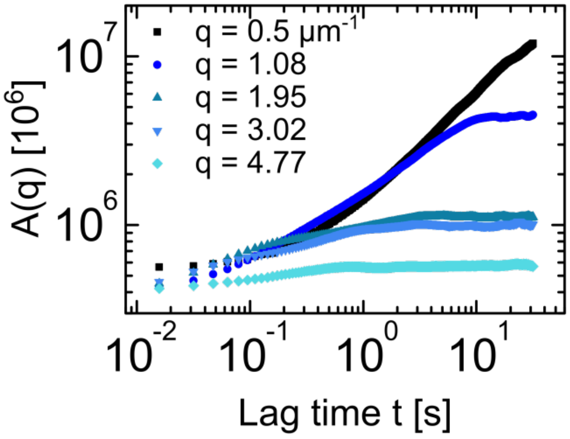

Supplemental Figure S1. Image structure function $A(q)$ as a function of lag time t for 5 different wavevectors ranging from 0.5 to 4.77 μm^{-1} for a bidisperse suspension of small (50 nm) and large (1 μm) particles at volume fraction ratio $r = \phi_L / \phi_S = 0.003$ and small particle volume fraction $\phi_S = 10^{-3}$.

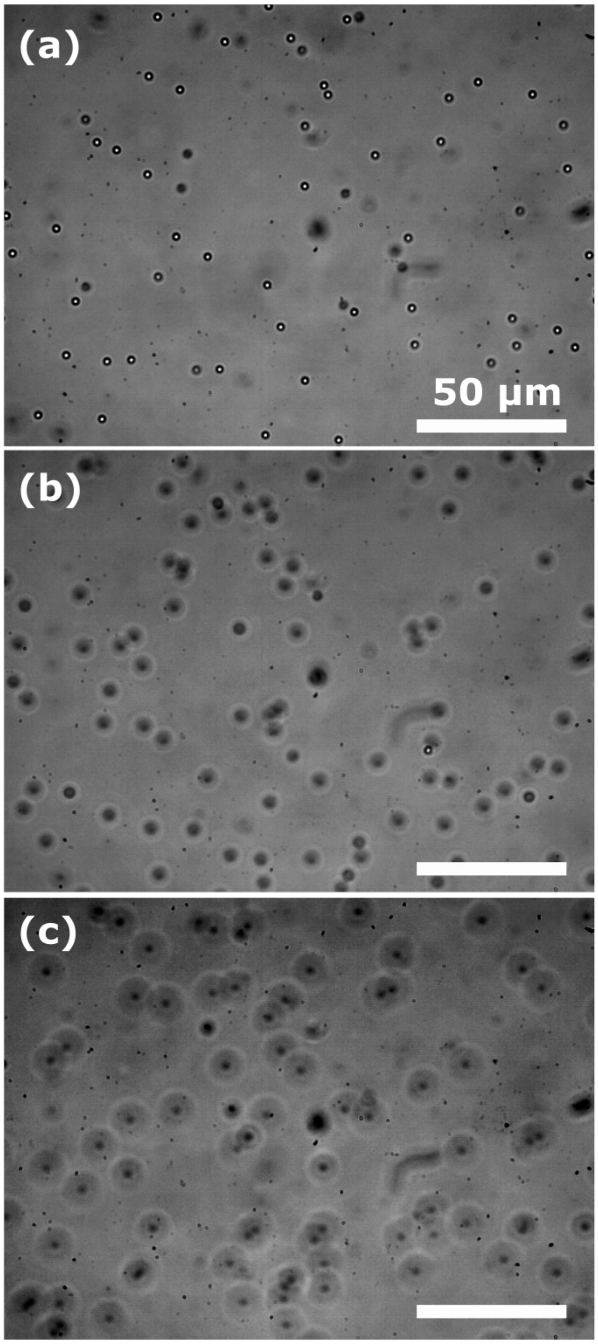

Supplemental Figure S2. Representative brightfield micrographs of particles in a unary sample with large particle volume fraction of $\phi_L = 10^{-5}$ that were segregated to the bottom of a glass sample chamber via sedimentation. Images were acquired at positions (*z*) of (a) 0, (b) 10 µm, and (c) 20 µm from the segregated particle plane. Scale bar is 50 µm.

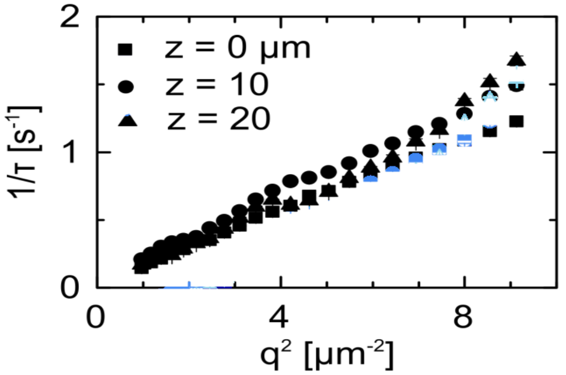

Supplemental Figure S3. Inverse of the large-particle time scale τ_L^{-1} as a function of the square of the wavevector q^2 for monodisperse suspension of large particles at a volume fraction of $\phi_L = 10^{-5}$ in sedimentation experiments at three different *z* heights from the segregated particle plane.

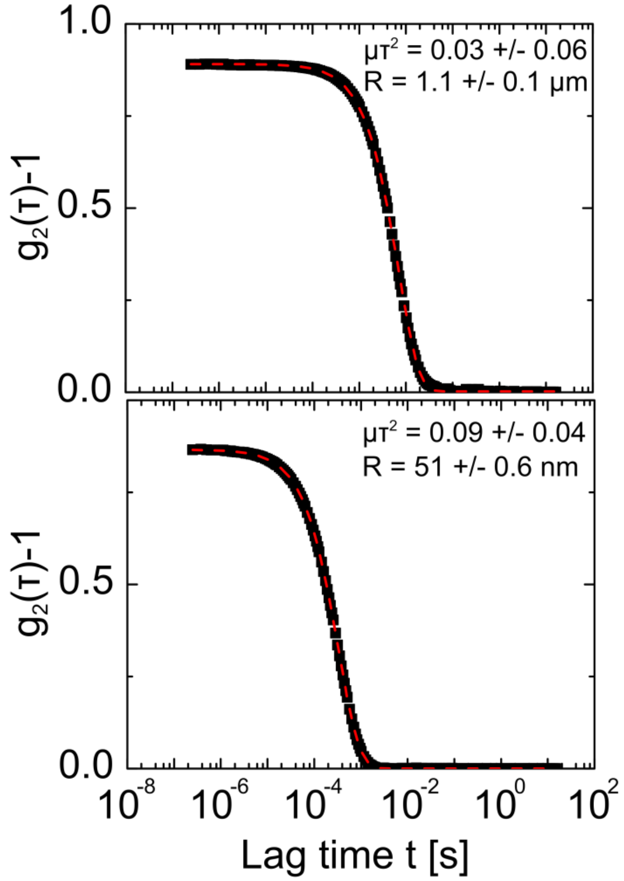

Supplemental Figure S4. Intensity correlation function g_2-1 as a function of lag time t for monodisperse suspensions of (a) small particles (radius 50 nm) at volume fraction of $\phi_S = 10^{-3}$ and (b) large particles (radius 1 μm) at a volume fraction of $\phi_L = 10^{-5}$. The polydispersity is determined from the second cumulant of the correlation functions fits. Measurements were performed at an angle of 90° (wavevector q = 18.7 μm^{-1}).
